# Induced Brain Plasticity after a Facilitation Programme for Autobiographical Memory in Multiple Sclerosis: A Preliminary Study

**DOI:** 10.1155/2012/820240

**Published:** 2012-10-18

**Authors:** Alexandra Ernst, Anne Botzung, Daniel Gounot, François Sellal, Frédéric Blanc, Jerome de Seze, Liliann Manning

**Affiliations:** ^1^Imaging and Cognitive Neurosciences Laboratory (CNRS UMR 7237, IFR 037), University of Strasbourg, 12 rue Goethe, 67000 Strasbourg, France; ^2^Colmar University Hospitals, Colmar and INSERM U-692, University of Strasbourg, 4 rue Kirschleger, 67085 Strasbourg, France; ^3^Neurology Unit, Strasbourg University Hospitals, 1 Av Moliere, 67098 Strasbourg, France; ^4^Clinical Investigation Centre, Strasbourg University Hospitals, 1 Av Moliere, 67098 Strasbourg, France

## Abstract

This preliminary study tackles the assessment and treatment of autobiographical memory (AbM) in relapsing-remitting multiple sclerosis (RR-MS) patients. Our aim was to investigate cerebral activation changes, following clinical improvement of AbM due to a cognitive training based on mental visual imagery (MVI). We assessed AbM using the Autobiographical Interview (AI) in eight patients and 15 controls. The latter subjects established normative data. The eight patients showed selective defective performance on the AI. Four patients were trained cognitively and underwent pre- and post-AI and fMRI. The remaining four patients took a second AI, at the same interval, but with no intervention in between. Results showed a significant improvement of AbM performance after the facilitation programme that could not be explained by learning effects since the AI scores remained stable between the two assessments in the second group of patients. As expected, AbM improvement was accompanied by an increased cerebral activity in posterior cerebral regions in post-facilitation fMRI examination. We interpret this activation changes in terms of reflecting the emphasis made on the role of MVI in memory retrieval through the facilitation programme. These preliminary significant clinical and neuroimaging changes suggest the beneficial effects of this technique to alleviate AbM retrieval deficit in MS patients.

## 1. Introduction

Multiple sclerosis (MS) is a neurological disease characterised by the multifocal nature of neurological lesions in the central nervous system, which results in demyelination, white and grey matter injuries and/or atrophy [1]. As a consequence of the cooccurrence of lesions, a wide range of symptoms can be observed in MS, including cognitive impairment [2]. Deficits on cognitive domains as anterograde memory, executive functions, attentional processes, or information processing speed have been described [3] and a number of clinical studies have also developed different cognitive rehabilitation programmes for MS patients [4].

However, very few studies have been carried out in MS to investigate autobiographical memory (AbM). Briefly stated, AbM is the capacity to relive detailed events, evoking the spatiotemporal context, in which they were encountered, as they are remembered [5]. AbM deficit in MS has been debated in the literature despite the comparatively modest number of works on the topic. Paul et al. [6] found personal semantics impairment in MS patients, while Kenealy et al. [7] observed episodic personal memory deficits, but these studies did not control for the illness subtypes. More recently, Müller et al. [8] reported impaired AbM in MS patients in secondary progressive MS patients and no deficit in relapsing-remitting multiple sclerosis patients (RR-MS), while Ernst et al. [9] demonstrated AbM deficit in RR-MS patients. Although, no “direct” comparison can be drawn between these two recent studies (Müller et al. and Ernst et al.), due to several methodological discrepancies, the contradictory findings are probably only apparent (see the Discussion section). Our previous clinical study [9] has shown that AbM impairment in RR-MS patients was very likely caused by a deficit of retrieval strategies, and that AbM deficit in MS could be alleviated with a cognitive facilitation programme. This programme was based on the cueing role of mental visual imagery (MVI) in AbM, which is involved in the reconstructive process of past events and allows to cue visual and other sensory modality information about the event [10, 11].

The clinical conclusions of our previous work led us to consider the cerebral substrates of the documented improvement of AbM functioning following the facilitation programme.

Several fMRI studies in MS have been reported, demonstrating cerebral activations during different cognitive tasks, particularly attentional processes and working memory tasks [12–14]. Those studies have highlighted the presence of a spontaneous cerebral plasticity in MS. Nevertheless, only a few studies have explored the possibility of an induced cerebral plasticity in MS patients following cognitive rehabilitation. These investigations on training-induced brain plasticity in MS have effectively shown cerebral activations changes after cognitive rehabilitation for attentional deficit, [15] attentional, dysexecutive and information processing impairments [16], or anterograde memory impairment [17]. No such study has been realised on AbM in MS patients, to our knowledge.

Therefore, the aim of the present preliminary study is to better document the mechanisms sustaining the efficacy of our MVI-based facilitation programme on AbM retrieval. To this end, we test both clinical and cerebral network changes before and after facilitation. Two sets of studies on neurocognitive relationships are relevant in the present work. In the first place, the studies showing that the AbM cerebral network is widespread and recruits predominantly the left and medial cerebral regions. Among those regions, the “core” structures are the prefrontal cortex, medial and lateral temporal cortices, parieto-occipital regions, temporoparietal junctions, and cerebellum [18]. Concerning prefrontal regions, several authors have pointed out their central role in the retrieval process [19, 20]. The further group of relevant studies in the present work are those reporting that MVI relies mostly on posterior cerebral regions [18] and that MVI implication in AbM is also supported by the partial overlapping of cerebral activations observed during AbM and MVI tasks [21]. On those bases, we hypothesised that the patients showing an improvement of AbM performance documented by clinical changes measured after cognitive training, would also show cerebral activation changes during the remembering processes of personal past events. More specifically, we expected an increased activation of posterior cerebral regions in the postfacilitation fMRI session given that our programme was constructed to enhance and optimise the role of MVI in AbM.

## 2. Materials and Methods

### 2.1. Participants

Eight patients with definite MS according to the Mac Donald criteria [22] were recruited at the Neurology Units of two French hospitals (Strasbourg and Colmar). Inclusion criteria were the diagnosis of a relapsing-remitting disease course, a mild functional disability corresponding to an Expanded Disability Status Scale (EDSS) [23] score ≤ 4, an absence of major signs of depression according to the Montgomery and Asberg Depression Rating Scale (MADRS; significant clinical threshold score ≥ 15), [24] no recent exacerbation of MS symptoms and right-handedness for patients who underwent fMRI sessions.

Fifteen healthy controls were also recruited to constitute a normative database for the AbM test. Exclusion criteria for all the participants included documented psychiatric illness, neurological disorder (other than MS for the patient group), and poor knowledge of French. Demographic and clinical data for the groups of MS patients are summarized in Table 1. All subjects signed prior informed consent, we complied with the APA ethical standards, and the research was approved by the “Committee for Protection of Persons” (CPP/CNRS N° 07023).

### 2.2. Neuropsychological Baseline Examination

The following baseline tests were presented to the MS patients. *Verbal IQ* was assessed with the short form [25] of the WAIS-III Verbal scale [26] and *nonverbal reasoning* with the Advanced Progressive Matrices Set 1 [27]. *Anterograde memory* was examined using the Rey auditory verbal learning test (RAVLT) [28] for the verbal modality and the Rey-Osterrieth Complex Figure (ROCF) [29, 30] was used to assess the visual modality. *Executive functions* were assessed using the phonological and categorical fluency tests (National Hospital, London), the Brixton Spatial Anticipation test [31], the Tower of London [32] and the Cognitive Estimation Task [33]. Concerning attentional and information processing, the *Information Processing Speed* test from the Adult Memory Information Processing Battery (AMIPB), [34] the Stroop test [35] and the months back test (National Hospital, London) were also administered. Since the experimental test assessing AbM is language-dependent, *language *was also examined, by means of the Déno 100 test [36]. The *visuoperceptual and visuospatial abilities* were tested using the Silhouettes and Cube Analysis subtests from the Visual Object and Space Perception Battery (VOSP) [37]. Finally, the short version of the “Echelle de Mesure de l'Impact de la Fatigue” (EMIF-SEP) [38] was proposed to evaluate the *impact of fatigue* in daily life.

### 2.3. Autobiographical Memory Assessment

The autobiographical interview [39] (AI; kindly communicated by the author, Brian Levine to one of us, LM.) was translated into French and adapted following Addis and colleagues [40]. Three cue-words were presented to the participants to probe past events for each of the four or five life periods depending on subject's age; 0–11 years, 12–20 years, 21 to (current age − 1) or 21–35 years, 36 to (current age − 1) and the previous year. We therefore obtained either 12 or 15 past events per participant, which differs from the usual AI procedure in which only one memory per life period is required. However, if only one single event per period was to be found, the participant was more likely to provide the most accessible detailed recollection [41, 42]. Consequently, we departed from the standard procedure to test a greater number of recollections in order both to probe more comprehensively the retrieval process and assess the benefits of our facilitation programme. The order of presentation of the cue-words and time periods was counterbalanced. Participants were asked to retrieve unique past events temporally and contextually specific, having occurred over minutes or hours (but not more than one day) and to generate freely as much details as possible about this event. They were informed that the cue-words, as stimuli triggering personal recollections, were intended to be used flexibly rather than literally. No time limit was set to avoid the potential influence of decreased cognitive processing speed on AbM performance. General probes were used to clarify instructions if necessary and to encourage the recall of additional details. The next step consisted in administering the specific probe phase, that is, a structured interview to test further details about a given event.

The assessment session was audio-recorded for later transcription and scoring. We carried out the latter using the AI standardised scoring procedure. Only two phases of recall were distinguished, that is, the free recall and specific probe phases, since the free recall and the general probe phases were analysed as a whole considering the minimal effect of general probes on recall [39]. However, scores were analysed cumulatively across the two levels of recall (i.e., free recall and specific probes). The first step of scoring was the identification of the main event, this permitting to classify each detail as internal (i.e., an episodic detail related to the main event) or external (i.e., nonepisodic information as semantic detail, repetition, metacognitive statement or episodic details but not related to the main event). A qualitative assessment of the episodic re-experiencing was also provided by ratings for episodic richness, time, space, perception and emotion/thought composites for each memory.

After an extensive training in the scoring method (also supplied by Brian Levine to one of us, L. Manning), scoring for all the participants was done by one scorer (A. Ernst) and 10% of the memories were analysed by a trained second scorer (five trained students), following Levine et al.'s instructions [39]. Coefficients for all measures showed a high interrater reliability (between 0.82 and 0.99).

We used a semistructured interview to obtain a qualitative evaluation by the patients of their AbM performance. The aim of this interview was twofold to characterise more precisely the patient's AbM difficulties as well as the potential effects of those difficulties in real life and to evaluate the benefits of the facilitation programme. This interview was adapted from the Memory Experiences Questionnaire, [43] from which we selected four dimensions (vividness, accessibility, sensory details and emotional intensity) based on our previous study [9]. A semi-structured interview was deemed to be better adapted than a questionnaire to explore everyday life.

### 2.4. Autobiographical Memory Facilitation Programme

The cognitive facilitation programme was constructed to alleviate AbM retrieval difficulties by means of a memory facilitation technique based on MVI. This technique was presented to the patients as a “tool” to facilitate access to a given recollection and the details associated to it. Therefore, during the MVI training sessions, patients found it increasingly easier to pay close attention to details in the mind's eye, which was used spontaneously when evoking personal recollections. Moreover, they were encouraged to try and use this technique in their everyday life and if needed, to ask questions to the neuropsychologist about the use of this tool. Treatment receipt, that is, “the extent to which the patient understands the strategies or techniques taught, and demonstrates the capacity to use them” (p. 836) [44] was verified at different times during the training process, by means of direct questions.

The programme consisted in 2-hour individual sessions once a week for at least six weeks. The patients performed MVI tasks of increasing difficulty, the content of which was divided into four steps. The rate of progression through these steps was flexible and could be adapted to each patient. Completion of the entire programme required at least six sessions but additional sessions were proposed if necessary to complete the programme at the patient's rhythm of training. All over the duration of the training programme, the role of the neuropsychologist was to teach how to construct visual scenes, and to provide continuous guidance throughout the training sessions.

1. The *screening test* was based on three sub-tests of the imagery and perception battery [45]: the mental representation of physical detail test, the morphological discrimination test, and the colour comparison test. We used a shortened version of each test with normative data established with a group of 15 healthy controls (different from the healthy control group who participated in the present study; unpublished data). We used these tests to probe basic visual imaging abilities, which enabled us to exclude patients presenting with severe MVI impairment (incompatible with the realisation of the facilitation programme). Thus, patients who presented scores below the normal range for all the three sub-tests were excluded.
2. The *external visualization* exercise included 10 verbal items that the patient had to imagine and describe. (We called this step “external” visualisation because the purpose of the exercise was to imagine an object and not the participant him/herself). The instruction ran as follows: “I will tell you the name of something (e.g., an onion), imagine it and describe its colour, shape, size, consistency and every detail that you see.” Each item description was checked for accuracy using a list of typical traits. For each item, the patient had to describe static aspects (e.g., colour, shape) and an action carried out with the item (e.g., slicing an onion). The action was imagined to be performed either by him/herself or someone else (no instruction was given as to who was supposed to act, since the patient was asked to concentrate on an object having changed in its physical appearance owing to an action).
3. The *construction* exercise of the programme consisted in figuring out complex scenes bringing into play several characters. The goal was twofold: to increase the complexity of the scene by comparison with the previous step and to introduce several characters performing different complex actions. Five items were proposed with, for each one, a first training step and a subsequent scene construction step. During the training phase, a neuropsychologist guided the patient, starting from a general idea (e.g., “a cook prepares a meal”), to more precise subheadings (e.g., “the cook is in front of a metallic table”). Within subheadings, detailed items were distinguished (e.g., “on the table, you can see butter, some onions and mushrooms …”). The present step was considered to be external since the participant was asked to imagine some characters and focus primarily on them, their actions and the context of the scene rather than the patient him/herself. Responses were checked for accuracy in terms of their relation to each subheading, but no list of typical or expected responses was used. The number of headings and subheadings, as well as details produced by the patients was recorded. For the scene construction phase, an item similar to the one used in the training section was proposed (e.g., “can you imagine the job you would most enjoy? Will you describe it, as we just did with the example of the cook?”).
4. The *self-visualisation* exercise was the training phase that shared the most similarities with AbM since the patients were asked to visualise themselves within a given scenario, to imagine it as though they were actually living the scene and to describe whatever details, sensations or feelings came to mind. The presentation was similar to that of the previous exercise, comprising a training phase (e.g., “you are heading towards a hotel reception desk”) and a scene construction phase (e.g., “your room is not ready; imagine yourself in the hotel bar”). The difference was that the participant was asked to focus his/her attention on him/herself, that is, using internal, personal knowledge. As for the previous step, we recorded the number of headings, sub-headings and details produced by the patients.

## 3. Imaging

Images were acquired using a 3T MRI scanner (Siemens Verio). The image sequence was a T2*-weighted echo planar imaging (EPI) sequence (TR = 2500 ms, TE = 30 ms, Matrix = 64 × 64 voxels, FOV = 224 mm, FA = 90). The anterior commissure (AC) and posterior commissure (PC) were identified in the midsagittal slice, and 45 contiguous slices (each 4 mm thick) were prescribed parallel to the AC-PC plane to cover the whole brain.

Concerning fMRI tasks, each of them was developed in two versions, randomly allocated for each patient to before and after facilitation sessions. The *experimental task* (i.e., past events condition) consisted in the evocation of unique personal past events temporally and contextually specific, occurring over minutes or hours, but no more than one day. Thirty-two pairs of words were proposed to elicit memories (e.g., purchase-car; country-walk), covering the same life periods than previously mentioned in the AI. Based on Addis et al., [46] two phases were distinguished in the evocation of the event: (i) the construction phase, which is the search and initial building up of the event, and (ii) the elaboration phase, which corresponds to the retrieval of details associated to the event. Each trial had a fixed duration of 20 s but the duration of the two phases, respectively, were determined by the patient's response: once a memory was retrieved, the patient had to press on the button 1 and this ended the construction phase. The remaining time, during which a central fixation cross was presented, was devoted to the elaboration phase. This was followed by a rating phase with two 4-point scales per event, each presented for 4 s, for which the patient had to rate the degree of vividness (1 = very poor vividness to 4 = very high vividness) and of emotional intensity felt during the remembering phase (1 = very poor emotional intensity to 4 = very high emotional intensity).

The *control task* was a categorical task that included 32 pairs of words, with which patients had to construct a sentence for the construction phase (e.g., with leather–boots: “In the shop, there are a lot of different leather boots”). Once the sentence was constructed, they had to press on the button 1 to pursue with the elaboration phase during which patients had to repeat the same sentence and to replace only the two initial words by words of the same semantic category (e.g., with the previous sentence: “In the shop, there are a lot of different cloth sneakers”; “In the shop, there are a lot of different plastic sandals”; etc). The fixed duration for each trial was also 20 s. Two 4-point scales, each for 4 s, followed each trial and patients had to assess the degree of difficulty (1 = very few difficulty to 4 = very high difficulty) and the degree of pleasantness (1 = very poor pleasantness to 4 = very high pleasantness). In both tasks the last rating was followed by short periods of fixation that were of jittered duration (mean duration = 1.5 s, range = 1 to 2 s).

It is to note that in the context of this preliminary study we focused our subsequent analyses on the construction phase of AbMs, since it reflects the access to AbMs, which according to our main hypothesis is defective in MS patients. Also, due to the small number of participants, we did not use vividness and emotional ratings for further analyses.

The experimental design was organised in four sequences of eight stimuli, beginning systematically with the control task and then alternating between past events and control task conditions. At the beginning of each sequence, the name of the condition was displayed on the screen for 6 s. The presentation order of stimuli within each condition was randomised.

The programming and responses collection was done with E-Prime 2 software (Psychology Software Tools, Inc.) and responses were given on a MR-compatible four-button response box. Words were displayed on a screen in white text with a black background and viewed using a mirror incorporated in the head-coil.

Prior to scanning, the tasks were explained to patients and they underwent a computerised practice trial for each task in order to be familiar to the experimental design and to the rhythm of presentation of the stimuli. They also received a more specific practice trial for the control task during which they were instructed to avoid self-implication in the sentences that they had to construct (e.g., no sentences with “I …”) and to try and minimise mental visual associations with the words, since these two processes were of interest for the experimental task.

Immediately following scanning, a postscan interview was realised for the past events condition in order to verify the adequacy of the responses and to exclude potential nonepisodic memories before the data analysis. Thus, for each past event, patients had to indicate the type of memory (unique, repetitive, extensive, semantic, or absent) and briefly, the spatial and temporal context of the event.

## 4. Procedure

The first section of the present study aimed at providing normative data for the AI. Consequently, the healthy control group underwent a single AI assessment session. The good matching between the group of healthy controls and MS patients was verified for age (*U* = 42.00;  *P* = 0.24) and gender distribution (*χ* ^2^ = 0.28;  *P* = 0.59). Mean scores of the number of internal details and total ratings were calculated for each recall phase and used to determine the normative data. The presence of an AbM impairment for our MS patients was determined using two measures. (i) The scores for the free recall phase owing to the free recall sensitivity to detect AbM retrieval deficit, which seems to be the memory process most involved in AbM impairment in MS patients. (ii) The mean number of internal details, which was 22.12 and total ratings, which was 8.38. These two measures assess the episodic reexperiencing ability and consequently, our MS patients' AbM performance was considered to be impaired if the mean score for internal details was ≤22.12 and the mean score for total ratings was ≤8.38 for the free recall phase.

Prior to inclusion, MS patients underwent a two-part screening tests. (i) The neuropsychological baseline examination (described above) to control for the absence of severe cognitive impairment other than AbM deficit. To pursue with the second part, patients had to be in the normal range on all tests (threshold: either  *z*-score −1.65 or the 5th percentile, depending on normative data) except for attentional and executive functions for which mild impairment was accepted. (ii) The AbM assessment session was carried out. To be included in the study, patients had to show AbM scores below the healthy controls' mean scores as mentioned above for internal details and total ratings for the free recall phase.

**Figure 1:**
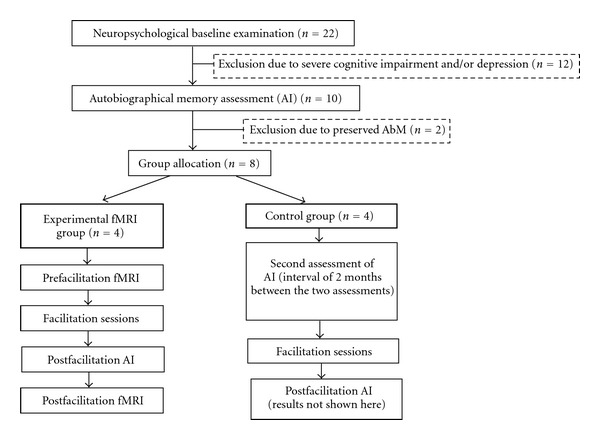
Diagram of the study design and progression trough study phases.

The patients included in the study were allocated in two groups: the experimental fMRI group and the control group. The allocation was not entirely randomised due to three patients, who were left-handed and could not be included in the experimental fMRI group. Patients in the experimental fMRI group underwent the MVI facilitation programme described above with two fMRI sessions following a before/after facilitation design. The effects of the facilitation programme on the behavioural measure were also assessed with the AI at the end of the programme. The control group was included to investigate potential learning effects on the second AI assessment session. Therefore, this group carried out the AI twice, with no intervention between the two sessions. However, for ethical reasons, the MVI facilitation programme was proposed to all the patients from the control group following the second assessment (results not shown here). A diagram summarising the study design and progression trough the study phases is presented in Figure 1.

The blind allocation of the patients' group could not be done since the baseline examination, the AbM assessments and the facilitation programme were conducted by the same neuropsychologist (A. Ernst). Nevertheless, to control a potential influence of the investigator awareness of the patients' group allocation, the second AI scorer was blind of the group membership, in every case. Moreover, AI reports were anonymised and memories were not supplied for scoring in the chronological order of assessment (i.e., after facilitation AI from a patient was not systematically given for scoring after the before facilitation AI).

## 5. Statistical Analyses

### 5.1. Behavioural Data

Potential differences between the two groups of MS patients for all tests of the neuropsychological baseline assessment were analysed with the Mann-Whitney test. Concerning the AI data, the Mann-Whitney test was used to detect between-group differences for each measure (i.e., mean number of internal details and the mean total ratings) for the free recall and specific probe phases. These comparisons were carried out for the two assessment sessions. Moreover, for each measure and recall phases mentioned above, a descriptive comparison between the patients' mean scores and the normative data from the healthy controls was carried out for the two AI assessments. A within-group analysis was also realised, by means of the Wilcoxon matched pairs test, to measure the degree of AbM improvement/stability between the two assessment sessions for the experimental fMRI and the control group. This procedure was used for the number of internal details and the total ratings for the free recall and specific probe phases.

### 5.2. Neuroimaging Data

Preprocessing and statistical analyses were conducted using SPM5 software (Wellcome Department of Cognitive Neurology, London, UK) [47]. Time-series were realigned to the first volume to correct for motion artefacts, spatially normalised to a standard EPI template based on the Montreal Neurological Institute (MNI) reference brain in Talairach space, [48] and then spatially smoothed using an 8 mm full-width at half-maximum isotropic Gaussian kernel. For both conditions and fMRI sessions, evoked hemodynamic responses time locked to the onset of the cue presentation (construction phase) were modelled with a canonical hemodynamic response function, and hemodynamic activity related to elaboration was modelled with a boxcar function of 10 sec-duration. Although all trials were modelled, only memory trials corresponding to unique past events (based on participants' responses during scanning and post-scan interviews) and correct control trials were included in regressors of interest. The ratings were modelled as a variable of no interest. Statistical parametric maps were generated for the comparison between the construction phase of past and control conditions (past versus control), for each subject, in the context of the general linear model. The two sets of contrast images (before and after facilitation) were then subjected to a second level of analysis to perform a within-subject comparison between before and after facilitation data (paired  *t*-test). For all these analyses, due to the small sample of patients, the significance threshold was set at *P* = 0.01, uncorrected for multiple comparisons, with a minimum extent threshold of 20 contiguously activated voxels.

## 6. Results

### 6.1. Behavioural Results

No significant difference was observed between the two groups of patients for the neuropsychological baseline examination, except for the MADRS, for which a higher mean score was observed for the control group (Table 2). However, the MADRS mean score for the two groups remained noticeably under the significant clinical threshold (score ≥ 15 to consider the presence of mild depression), consequently, this difference between the two groups was not taken into account in further analyses. Neuropsychological baseline's results showed impaired performances in only one attentional test for one patient in each group and low-average scores for one or two tests exploring executive functions for 3 patients of the experimental fMRI group and 2 patients of the control group.

Turning to AbM performances, for the first AI assessment, the mean number of internal details and the mean total ratings obtained during the free recall phase were 18.07 and 5.58, respectively, for the experimental fMRI group and 19.22 and 5.72, respectively, for the control group. For both groups, the scores were below the mean scores established with the healthy controls (see above). In regard to the *first* AI assessment, no significant difference for the mean number of internal details (*U* = 8.00;  *P* = 1.00) nor the mean total ratings (*U* = 5.00;  *P* = 0.38) during the free recall phase were observed between the two groups of patients. The same results were obtained for the specific probe phase (mean number of internal details:  *U* = 7.00;  *P* = 0.77 and mean total ratings:  *U* = 6.00;  *P* = 0.56). Likewise, the patients' mean scores for the specific probe phase (39.33 internal details for the experimental fMRI group and 39.00 internal details for the control group, with a mean total ratings of 11.12 for the experimental fMRI group and 10.58 for the control group), were below those obtained by the healthy controls (40.2 for the mean number of internal details and 12.47 for the mean total ratings).

In contrast, the scores on the second AI assessment showed significant differences between the experimental fMRI and the control groups for the free recall phase (mean number of internal details:  *U* = 0.00;  *P* = 0.02  and mean total ratings:  *U* = 0.00;  *P* = 0.02) and for the specific probe phase (mean number of internal details: *U* = 0.00;  *P* = 0.02 and mean total ratings:  *U* = 0.00;  *P* = 0.02). In every case, patients included in the experimental fMRI group performed significantly better than those in the control group during the second AI assessment, which corresponded to the post-facilitation examination for the experimental fMRI group.

With regard to the experimental fMRI group, the within-group analysis for before and after facilitation AI assessment highlighted a significant improvement of the patients' scores on the AI for all the measures of the free recall phase (number of internal details:  *T* = 104.50;  *P* = 0.00 and total ratings: *T* = 95.00;  *P* = 0.00) and of the specific probe phase (number of internal details: *T* = 81.00; *P* = 0.00 and total ratings: *T* = 155.50; *P* = 0.00). Improvement of AI scores for the experimental fMRI group after facilitation condition was supported by the comparisons with those of the healthy controls. Indeed, the patient group showed a normalisation of their mean score for all the free recall phase measures (mean number of internal details = 36.9 versus 22.12 and mean total ratings = 9.39 versus 8.38) and for the specific probe phase (patients' mean number of internal details = 68.85 versus 40.2 and mean total ratings = 14.79 versus 12.47). Conversely, the comparison of the two sessions of AI assessment for the control group showed no significant differences during the free recall phase for the number of internal details (*T* = 622.50; *P* = 0.70) nor the total ratings (*T* = 557.00; *P* = 0.94). Also during the specific probe phase, no significant differences were observed for the number of internal details (*T* = 571.50; *P* = 0.39) nor the total ratings (*T* = 545.00; *P* = 0.65). For the second AI assessment of the control group, the results of the comparisons with the healthy controls' mean scores were parallel to their first AI examination, including mean scores below those of the healthy controls for the free recall phase (mean number of internal details = 20.6 versus 22.12 and mean total ratings = 5.83 versus 8.38). For the specific probe phase, an “apparent normalisation” was observed for the mean number of internal details (patients, 42.12 versus 40.2). Qualitative analysis revealed a punctual variation that resulted in an increased score (namely, one recollection). This observation together with the absence of a significant difference for the mean number of internal details between the two AI assessments for the control group, led us to rule out a normalisation process in this case. Moreover, no improvement was observed for the mean total ratings relative to normal controls (11.08 versus 12.47).

Based on the semi-structured interview, the patients' comments confirmed the positive effects of the facilitation programme during the second AI testing as well as in everyday life. The perceived benefits concerned a greater easiness of retrieval, a greater amount of details and better vividness during memory evocation. However, no change was mentioned concerning the emotional intensity. An effective treatment receipt seemed to be obtained since the patients acknowledged an easy use and transfer of this technique in their daily life functioning. Additionally, we also had the opportunity to get spontaneous comments by some patients' relatives who confirmed the benefits of the facilitation programme.

## 7. Neuroimaging Results

### 7.1. After versus before Facilitation Data

**Figure 2:**
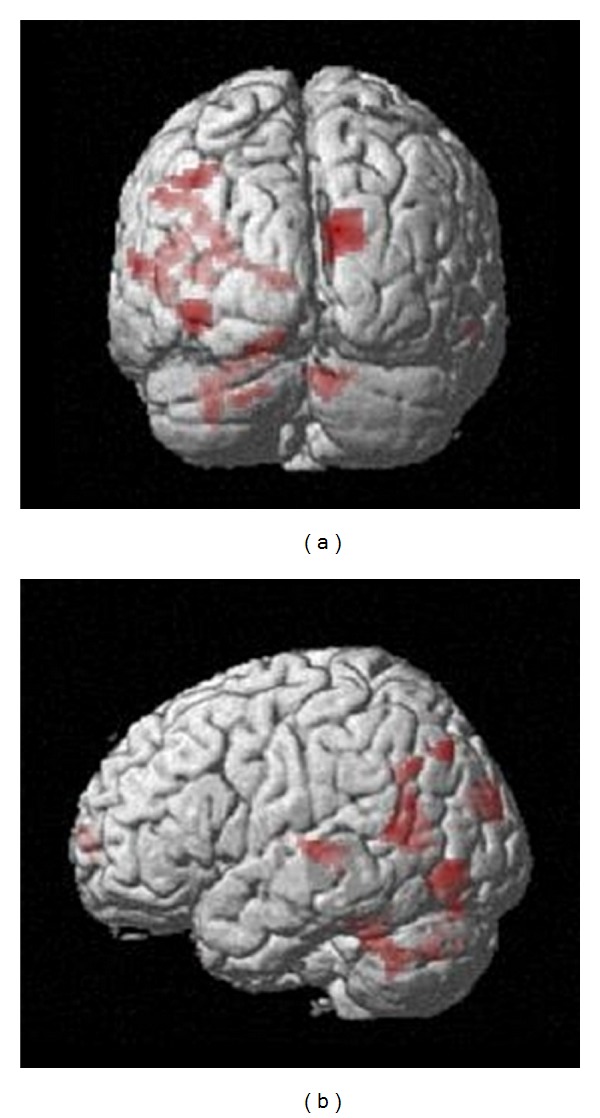
Posterior (top) and right lateral (bottom) views of the group activation map showing increased differential activity *after* the facilitation programme, for the contrast between past and control conditions (*P* = 0.01,  *k* > 20).

The paired *t*-test comparison we performed, for the contrast between past and control conditions, revealed that brain regions exhibiting a greater difference in activity between after and before facilitation fMRI sessions were primarily located in posterior regions. More precisely, after the facilitation programme, our patients elicited greater recruitment of the right cuneus (BA 19), the left inferior and superior occipital gyri (BA 18, 19), the left precuneus (BA 19) as well as of parts of the lateral temporal cortex, primarily on the left (BA 22, 38, 39) (see Table 3 and Figure 2).

### 7.2. Before versus after Facilitation Data

**Figure 3:**
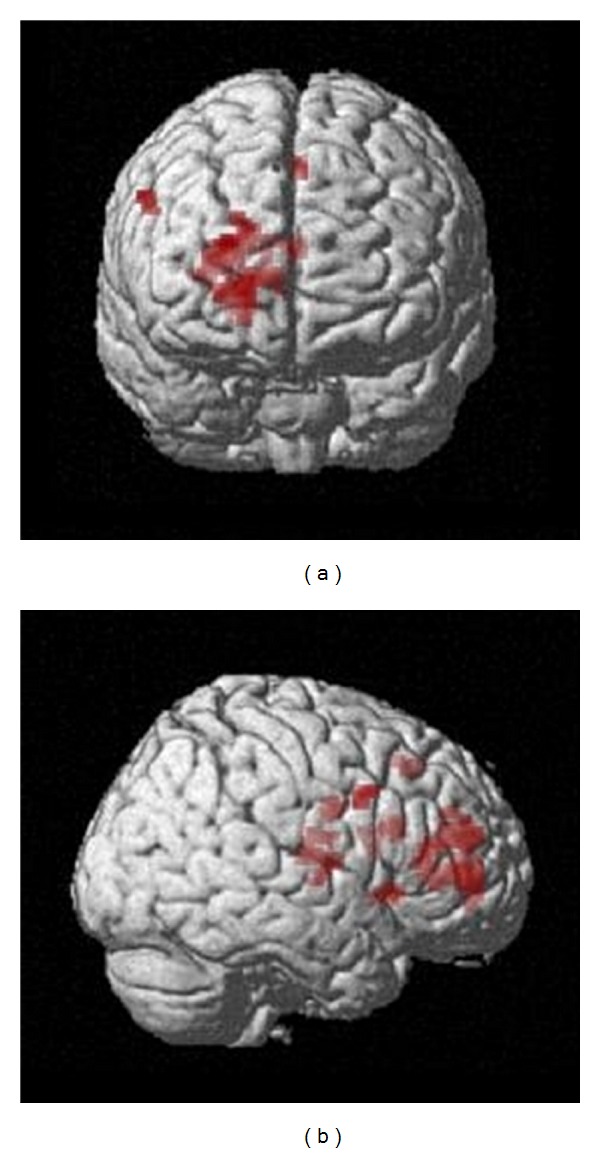
Anterior (top) and left lateral (bottom) views of the group activation map showing increased differential activity *before* the facilitation programme, for the contrast between past and control conditions (*P* = 0.01,  *k* > 20).

The reverse comparison indicated that regions showing a greater difference in activity before the facilitation programme were exclusively located in anterior parts of the brain: the two larger clusters of differential activity were indeed located in the prefrontal cortex (PFC), the first encompassing both dorsolateral and medial aspects of the PFC, the second corresponding to the dorsolateral PFC (BA 45, 9) (see Table 4 and Figure 3).

## 8. Discussion

In the present preliminary study, we reported AbM performance improvement following a facilitation programme in four RR-MS patients and provided evidence of changes at the neural level. The clinical improvement was verified on the AI [39], which entailed obtaining French normative data. Importantly, our results are comparable to those reported by Levine et al. [39]. Based on these normative data, we showed a normalisation of patients' AbM free recall performances following the facilitation programme, which suggested a compensation of the initial retrieval deficit. We controlled for the potential learning effects of the AbM test by means of comparisons between an experimental fMRI group and a control group comprising also four RR-MS patients, who received no cognitive intervention but underwent the AI test twice.

Besides our previous study, [9] only three further works, to our knowledge, have tackled AbM assessment in MS. Our findings are in accord with Kenealy et al. [7] and they are contrary to those obtained by Paul et al. [6] and Müller et al. [8]. These two latter studies concluded that episodic personal memory was preserved in their MS patients either with no specification of the illness subtypes [6] or in defined RR-MS subtype [8]. The test used in these two works was the autobiographical memory interview (AMI), [49] which has been said to have a good sensitivity for the personal semantics section, but a poor sensitivity for the episodic incident section [50, 51]. Interestingly, Müller et al. [8] also found that patients with secondary progressive MS exhibited graded loss of personal incident memory on the AMI. However, the absence of such AbM deficit for RR-MS in Müller et al.'s [8] study could suggest that the AbM impairment in RR-MS is moderate and subtle and consequently, not readily detected by the AMI. These results and what they suggest are coherent with our results using a more stringent AbM test in RR-MS patients, which show mild to moderate AbM deficit. Although this is the first study, to our knowledge, to use the AI in MS, it has already been used in different neurological conditions like temporal lobe epilepsy [52], Alzheimer disease [53] or semantic dementia [50], showing the effectiveness of this autobiographical test in clinical settings.

Moreover, our preliminary fMRI findings are coherent with our retrieval-deficit hypotheses, involving a frontal lobe dysfunction [19, 20], in the AbM deficit in MS. Indeed, the enhanced cerebral activation during the construction phase of past recollections was observed in the bilateral prefrontal regions, only during the before facilitation session. This observation suggests a bi-lateralisation of cerebral activations since the AbM core cerebral network involves predominantly left cerebral regions [18]. Furthermore, it could reflect a compensation mechanism attempt, although insufficient to result in normal AbM performance. This suggestion is supported by studies exploring spontaneous functional reorganisation in MS, which show compensation mechanisms during cognitive task, but which are not always powerful enough to result in a complete preservation of cognitive functioning and consequently give rise to mild cognitive impairment [13, 54].

With regard to our fMRI results following the facilitation programme, they are coherent with the published reports dealing with the effects of various cognitive rehabilitation programmes on neural substrates in MS patients, that is, the presence of cerebral activation changes after cognitive intervention [15–17].

The present fMRI findings are coherent with our hypothesis since they showed that the improved AbM performance was accompanied by cerebral activation changes during the construction processes of personal past recollections. Importantly, changes in activation were observed in posterior cerebral regions, which are known to be associated with the mental processes we trained in our MS patients, that is, MVI [18]. Moreover, these changes were observed during the construction phase, which indicates the effective use of MVI to retrieve memories. This latter finding is consistent, not only with the global improvement of AbM scores after the facilitation programme but also, and above all, with the normalisation of AbM scores for all the patients during the free recall phase, which is a sensitive indicator of retrieval deficit. The posterior cerebral regions showing activation changes were, probably, under activated secondary to an insufficient PFC activation (see above). This suggestion is based on research using hemodynamic response function (HRF) analyses during AbM construction processes. Indeed, an earlier peak in the PFC relative to peaks reached in more posterior regions has been shown [19]. In cases in which the initial PFC HRF is subnormal, the HRF curve of the following (more posterior) regions could be affected and present under activation. This would also imply a possibility of “return to normal” either by a normalised initial HRF peak or by compensation mechanisms induced by cognitive training.

Beside these quantitative changes, the differences between the before and after facilitation semi structured interviews of patients showed positive effects in terms of a greater easiness of retrieval, a greater amount of details, a better vividness and an effective use of this technique in their daily life functioning. In addition, some patients' relatives confirmed the benefits of the facilitation programme in daily life. These last points are of importance considering that the primary goal of cognitive rehabilitation is to improve everyday life functioning in patients [55, 56].

While these preliminary findings are of interest, we are aware of some methodological limitations that restrict the conclusions that can be drawn from this study. Despite the statistical evidence obtained for behavioural and fMRI data, our sample of patients is small and replication of these results with a larger sample would be required. Additionally, a larger sample could also give the opportunity to explore more precisely the link between the changes observed for clinical and fMRI measures. With regard to the facilitation programme, the presence of a “nursing effect” cannot be ruled out until a “placebo” group of patients is included. Furthermore, long-term follow-up measures would be valuable to examine the robustness of the treatment effects.

Notwithstanding these limitations, our preliminary findings seem to be promising and encourage further investigations, all the more so that our results are in agreement with findings from previous studies which have explored the effect of cognitive rehabilitation on behavioural and neuroimaging measures in MS in diverse cognitive functions (attention, [15] executive functions and information processing [16] or anterograde memory [17]).

Finally, this preliminary study is, to the best of our knowledge, the first neuroimaging study focusing on AbM cognitive facilitation in MS patients. Expanding this type of intervention for MS patients seems necessary due to the deleterious impact of cognitive deficit in everyday life. This is particularly the case with AbM, considering its importance in everyday life functioning [57, 58].

## Figures and Tables

**Table 1:** Demographical and clinical data (mean and standard deviation) for the two groups of MS patients.

	Experimental fMRI group	Control group	Statistical analysis
*N* =	4	4	—
Age in years	37.25 SD 5.50	39.75 SD 5.06	*U* = 6.50; *P* = 0.66
Education in years	12.75 SD 1.50	11.75 SD 0.50	*U* = 4.50; *P* = 0.31
Sex (ratio female/male)	3/1	2/2	*χ* ^2^ = 0.53; *P* = 0.465
Laterality (ratio right-/left-handed)	4/0	1/3	*χ* ^2^ = 4.8; *P* = 0.028*

EDSS (median score)	1.50	2.50	*U* = 7.50; *P* = 0.88
[range]	[0–4]	[0–4]	
Duration of MS in years	15.00 SD 9.31	13.5 SD 7.23	*U* = 6.50; *P* = 0.66
Number of DMD treatment	1.00 SD 0.00	1.00 SD 0.00	*U* = 8.00; *P* = 1.00

EDSS: the expanded disability status scale; SD: standard deviation; DMDs: disease modifying drugs, *<0.05.

**Table 2:** Scores (mean and SD) for all MS patients by group for the neuropsychological baseline examination.

	Experimental fMRI group	Control group	Statistical analysis
Verbal IQ	94.00 SD 13.04	93.25 SD 11.44	*U* = 7.00; *P* = 0.77
PM12	10.25 SD 0.96	8.25 SD 3.20	*U* = 6.00; *P* = 0.56

RAVLT			
Total mean number of words	12.40 SD 1.10	12.80 SD 1.42	*U* = 6.00; *P* = 0.56
Delayed recall	14.25 SD 0.96	14.25 SD 0.96	*U* = 8.00; *P* = 1.00
ROCF			
Copy	35.50 SD 1.00	35.75 SD 0.50	*U* = 7.50; *P* = 0.88
Immediate recall	27.25 SD 4.99	22.50 SD 1.22	*U* = 2.50; *P* = 0.11
Delayed recall	27.25 SD 4.19	24.38 SD 2.14	*U* = 4.50; *P* = 0.31

Deno 100	97.25 SD 3.77	98.25 SD 2.06	*U* = 7.50; *P* = 0.88

Stroop			
Colours (score *T*)	43.00 SD 10.49	43.75 SD 10.43	*U* = 7.00; *P* = 0.77
Words (score *T*)	44.50 SD 4.12	48.75 SD 5.85	*U* = 4.00; *P* = 0.25
Interference (score *T*)	45.50 SD 15.72	48.00 SD 12.57	*U* = 8.00; *P* = 1.00
Interference score (score *T*)	47.25 SD 12.04	51.50 SD 5.80	*U* = 6.50; *P* = 0.66
Months back (sec)	10.75 SD 4.50	11.75 SD 2.75	*U* = 6.00; *P* = 0.56

Tower of London			
Score	7.50 SD 1.73	9.00 SD 0.82	*U* = 3.00; *P* = 0.14
Time indice	21.25 SD 6.50	18.50 SD 1.29	*U* = 7.50; *P* = 0.88
Brixton (number of errors)	16.50 SD 3.11	13.50 SD 6.56	*U* = 6.00; *P* = 0.56
Cognitive estimation task	3.50 SD 3.11	4.25 SD 4.03	*U* = 7.50; *P* = 0.88
Verbal fluency			
Categorical	19.50 SD 3.87	19.00 SD 8.12	*U* = 5.00; *P* = 0.38
Phonological	11.25 SD 3.86	14.00 SD 4.69	*U* = 6.50; *P* = 0.66

Information processing speed			
Cognitive	52.75 SD 11.32	50.50 SD 10.15	*U* = 7.00; *P* = 0.77
Motor	40.25 SD 10.53	51.75 SD 10.44	*U* = 2.00; *P* = 0.08
Error percentage	3.29 SD 3.79	1.76 SD 1.36	*U* = 6.50; *P* = 0.66
Corrected score	59.96 SD 13.71	55.29 SD 11.90	*U* = 6.00; *P* = 0.56

VOSP			
Silhouettes	20.25 SD 2.63	21.75 SD 3.30	*U* = 5.50; *P* = 0.47
Cubes analysis	9.75 SD 0.50	9.75 SD 0.50	*U* = 8.00; *P* = 1.00

MADRS	2.50 SD 3.00	7.75 SD 2.87	*U* = 1.00; *P* = 0.04*

EMIF-SEP (total)	39.57 SD 8.12	42.56 SD 15.74	*U* = 4.50; *P* = 0.31

PM12: Progressive matrices 12; RAVLT: Rey auditory verbal learning test; ROCF: Rey-Osterrieth Complex Figure; VOSP: visual object and space perception; MADRS: Montgomery and Asberg depression rating scale; EMIF-SEP: Echelle de mesure de l'impact de la fatigue, *<0.05.

**Table 3:** Brain regions exhibiting a greater difference in activity during the after versus before rehabilitation fMRI session in the comparison between past and control conditions.

Brain Region	Coordinates (*x, y, z*)	*Z*-score	Cluster size
R Cuneus (BA 19)	(12, −94, 24)	4.62	179
L Inferior/Middle Occipital Gyrus (BA 18, 19)	(−36, −74, −4)	3.84	115
L Precuneus (BA 19)	(−36, −76, 44)	2.82	39
L Superior Temporal Gyrus/Middle Temporal Gyrus/Posterior Cingulate (BA 22, 39, 31)	(−60, −54, 12)	3.91	240
L Superior Temporal Gyrus (BA 41)	(−48, −22, 6)	3.59	66
R Middle Temporal Gyrus (BA 21)	(58, −2, −12)	3.31	21
R Superior Temporal Gyrus (BA 38)	(52, 18, −34)	3.25	22
R Superior Frontal Gyrus (BA 10)	(28, 64, 4)	3.31	47
R Thalamus	(12, −14, 12)	4.43	75
L Caudate/Thalamus	(−34, −32, 0)	3.05	29
L Cerebellum	(−34, −52, −32)	3.94	118
R Cerebellum	(8, −72, −28)	3.73	61
L Cerebellum	(−16, −68, −34)	3.63	25
L Cerebellum	(−16, −78, −20)	3.27	40

Talaraich Coordinates Reported. BA: Brodmann's area; L: left hemisphere; R: right hemisphere.

**Table 4:** Brain regions exhibiting a greater difference in activity during the before versus after rehabilitation fMRI session in the comparison between past and control conditions.

Brain Region	Coordinates (*x, y, z*)	*Z*-score	Cluster size
R Medial/Superior Frontal Gyrus (BA 10)	(16, 46, 8)	4.14	192
R Inferior/Superior Frontal Gyrus (BA 45, 9)	(34, 28, 10)	3.96	163
R Inferior Frontal Gyrus (BA 9)	(52, 4, 32)	3.37	23
R Anterior Cingulate (BA 33)	(2, 20, 18)	3.36	27
L Superior Frontal Gyrus (BA 8)	(−2, 26, 48)	3.04	29
R Insula (BA 13)	(40, −10, 2)	3.86	80
R Putamen	(20, 20, −4)	3.51	41
R Caudate/Cingulate Gyrus (BA 32)	(10, 10, 16)	3.39	54

Talaraich Coordinates Reported. BA: Brodmann's area; L: left hemisphere; R: right hemisphere.
